# Just my luck: Diagnosed difficult

**DOI:** 10.1002/jhm.70067

**Published:** 2025-05-11

**Authors:** Nike Izmaylov, Michelle Izmaylov

**Affiliations:** ^1^ Vanderbilt University Medical Center Nashville Tennessee USA

She frowns when I walk into the room, her eyes narrowed as she watches my approach. I pause a few deliberate steps away from her. The chart warned me that this patient would be difficult. The wariness in her stare, her tablet held defensively against her chest, appears to confirm that.

I stand at the bedside while talking to this woman with an undefined gastrointestinal problem that causes her significant pain. The chart informed me that she had seen many different gastroenterology doctors who had provided her with a variety of diagnoses and medications. She had been scoped repeatedly. She had been assessed by multiple experts who specialize in zebras.

Her apparent diagnosis is indicated by her chart: a patient seeking pain medications for functional pain, the plan for her admission to facilitate urgent discharge.

While we talk, I consider the warning of the prior residents. I was told not to go into her room and that we were spacing out her opioids so that she could leave without them, informed that this is really a psychiatric patient and there is nothing for medicine to do. My initial encounter appears to confirm this: when I tell her that we are planning to decrease her opiates, her tone starts to glint with jagged edges, and her husband gets up from the chair he'd been commanding. I step back, step away from the perception of a threat. But he ignores me and puts his hand on her shoulder.

I leave within a few minutes of going into that room. It appears the chart is correct: this is a patient who wants opiates, who discontinued the PPIs she had been prescribed, who is not compliant, who is going to be a challenge. For the next few days, I find an excuse to get out of her room within as few minutes as possible when we round.

That's just my luck during a busy day: a difficult patient.

It is not just the warnings that urge me to stay away: it is the intensity of the workload. Talking to a patient asking for opiates must necessarily be triaged to a lower priority compared with those with vital sign changes or paperwork needed urgently for discharge.

I learned this through working the cross‐cover shift, when pages emphasizing stable chronic pain were set aside to address patients with hypotension, with urgent labs to follow up from the day, with the sudden onset of abdominal pain prompting need for a consult. I learned this through the administrative barriers I had to surmount to ensure even compliant patients had prescribed opiates on discharge, the searches for seldom‐used prescription pads and push‐back from electronic medical records taking significant effort in a day with multiple other discharges pending.

I learned this through the mental tracks that develop one's scripts for patient encounters. This man with fever, rash, and elevated transaminases should be examined for ticks. This woman with confusion and leukocytosis should be checked for a urinary tract infection. This patient, who presents repeatedly to the emergency department asking for “that med that starts with a D” should be assessed for being a “drug seeker.”

The work of improving the opioid epidemic has made it difficult for patients who need medications to get treatment.

The work of managing the fallout has made clinicians, including myself, too jaded to spend sufficient effort to help these patients.

But when it's just my luck to have an afternoon of few admissions and acute issues, I consider that we haven't made progress toward discharge. My reason for approach is not to try to “understand her better,” but to continue the work of discharging patients as hospital policy and productivity metrics demand. Now that my lack‐of‐workload permits this conversation, I walk into her room to talk to her.

I approach her, and she glares at me with what is now suspicion. But maybe the reason is that I mostly ignored her for several days.

I pull up a chair, signaling an intent to stay for a longer discussion. I ask the patient how this started. She starts with a few words, pausing to study my responses, perhaps concerned that I am not listening. When she recognizes that I am paying attention, she tells me more.

I learn that she had been to the emergency department (ED) multiple times for pain crises and had been labeled as a drug seeker. She recognized that opiate medications were not the solution to her pain, and she did not push through the ED doors demanding such medications. Previously, she attempted many medications and treatments, including cognitive behavioral therapy (CBT) when there was concern that the pain was of psychiatric etiology. She was not opposed to CBT in principle, but she opposed this: the attempted gaslighting during the sessions, the therapist insisting that she was not having pain, and dismissing her concerns.

Many clinicians approached her in that manner, ignoring pain when the etiology could not be identified on imaging. Is not having a defined etiology sufficient reason to erase the pain that brought her to the hospital?

We continue to discuss her case. I had been told that the patient and her relatives were seeking One True Diagnosis with a Silver Bullet that would Fix Them. Yet, when I actually speak to her, without *any* prompting from my part, the patient expresses—on her own—that many different etiologies are contributing to her pain.

She recognizes that the medications she has been prescribed were trying to tackle these different contributory mechanisms. But the journey has been overwhelming to her, and the myriad of treatments have not actually made her feel better. I learn that she did not discontinue the PPI from intentional noncompliance, but rather that she did not know this medication might need several weeks to work. When I re‐explain this, she tells me that she does vaguely remember another physician telling her that, too, but she had heard so many things from so many different experts that it had been a kaleidoscopic blur before now. She wants to continue the PPI.

But then, she asks me: why did I think she was intentionally noncompliant with her medications? I tell her this is what I gathered from the documentation in her chart. I notice the impact of that statement how she shields herself with the tablet, though that tension is not directed at me. Rather, she recognizes that documentation pursues her through each hospitalization, and clinicians apply a label to her before meeting her.

From signout, I, too, had assumed she was a drug seeker, long before I ever spoke to her.

I tell her that I plan to include additional documentation detailing our discussion, including her reasons for not being on all the medications that had been prescribed. By the time the patient discharges, the team and I have not yet found a solution to the pain, yet she appreciates that I have initiated her on an oral solution of opiates that she finds less painful to take than the pills she had been given before, and more importantly that I found a moment to listen to her concerns.

But I did not deliberately find such a moment. I did not add an additional hour to my clinical day. At the typical pace of work, I would not have the availability for that conversation. It was just a day with many fewer patients and admissions than normal that allowed me this moment of connection.

When I go back to the workroom, I look through all the patients I briefly talked to during rounds. I—and many of my colleagues—want to have such a detailed discussion with each patient.

But there's another admission, another rapid response, and too few clinicians to provide that level of care for each patient.

A few weeks later, I notice that the patient with abdominal pain is once again in the ED. This, too, is just my luck: a coincidence of checking the ED board when I normally do not, having walked to the ED to see a different patient. She was not placed on my primary service, but I go talk to her. The patient tells me that she had difficulty getting the oral opiates from the specialty pharmacy, and she is here to troubleshoot those challenges. She is thankful for my care. When I leave the room, I speak briefly with the residents now providing her care and inform them that—even if they read previous notes of the patient being difficult and uncooperative—this patient was *not* those things when she was actually listened to.

Several weeks after that, one of the residents pauses in the hallway to let me know that he appreciated that I reached out to them.

When the new residents started reading over her chart, they dreaded having to take care of her, but my conversation prompted them to walk in prepared to listen instead of assuming that she would be difficult. This set a positive tone for the entirety of her hospital stay, and she has been discharged without any issue, hopefully with better access to the liquid medications that can control her pain.

What I brought to her care was *not* more compassion, *not* more concern for the patient I was treating, and *not* more medical knowledge than my colleagues. I was just the clinician who had happened to have an hour to talk to her during an afternoon in the otherwise understaffed and overworked environment of modern medicine—it was just my luck.

But that luck is what made all the difference to this patient's care.

## CONFLICTS OF INTEREST STATEMENT

The authors declare no conflicts of interest.

